# Automated ventricular systems segmentation in brain CT images by combining low-level segmentation and high-level template matching

**DOI:** 10.1186/1472-6947-9-S1-S4

**Published:** 2009-11-03

**Authors:** Wenan Chen, Rebecca Smith, Soo-Yeon Ji, Kevin R Ward, Kayvan Najarian

**Affiliations:** 1 Department of Computer Science, Virginia Commonwealth University, 401 East Main Street, Richmond, VA, USA; 2 Department of Emergency Medicine, Virginia Commonwealth University, 401 N 12th St, Richmond, VA, USA

## Abstract

**Background:**

Accurate analysis of CT brain scans is vital for diagnosis and treatment of Traumatic Brain Injuries (TBI). Automatic processing of these CT brain scans could speed up the decision making process, lower the cost of healthcare, and reduce the chance of human error. In this paper, we focus on automatic processing of CT brain images to segment and identify the ventricular systems. The segmentation of ventricles provides quantitative measures on the changes of ventricles in the brain that form vital diagnosis information.

**Methods:**

First all CT slices are aligned by detecting the ideal midlines in all images. The initial estimation of the ideal midline of the brain is found based on skull symmetry and then the initial estimate is further refined using detected anatomical features. Then a two-step method is used for ventricle segmentation. First a low-level segmentation on each pixel is applied on the CT images. For this step, both Iterated Conditional Mode (ICM) and Maximum A Posteriori Spatial Probability (MASP) are evaluated and compared. The second step applies template matching algorithm to identify objects in the initial low-level segmentation as ventricles. Experiments for ventricle segmentation are conducted using a relatively large CT dataset containing mild and severe TBI cases.

**Results:**

Experiments show that the acceptable rate of the ideal midline detection is over 95%. Two measurements are defined to evaluate ventricle recognition results. The first measure is a sensitivity-like measure and the second is a false positive-like measure. For the first measurement, the rate is 100% indicating that all ventricles are identified in all slices. The false positives-like measurement is 8.59%. We also point out the similarities and differences between ICM and MASP algorithms through both mathematically relationships and segmentation results on CT images.

**Conclusion:**

The experiments show the reliability of the proposed algorithms. The novelty of the proposed method lies in its incorporation of anatomical features for ideal midline detection and the two-step ventricle segmentation method. Our method offers the following improvements over existing approaches: accurate detection of the ideal midline and accurate recognition of ventricles using both anatomical features and spatial templates derived from Magnetic Resonance Images.

## Introduction

It is estimated that every year, 1.4 million people in the United States sustain a Traumatic Brain Injury (TBI) [1]. TBI occurs when a sudden event damages the brain, such as a blow or jolt to the head. Over 50,000 of these patients will not survive, and many others will be left permanently disabled [1]. 50% of those who die do so within the first two hours after injury [2]. Speed of diagnosis is therefore vital, and so Computed Tomography (CT) imaging, which is faster and much less costly than other medical scan, e.g., Magnetic Resonance Imaging (MRI) scan, is still the gold standard for initial TBI assessment [3]. A CT scan can also clearly reveal any severe abnormalities such as fractures or hematomas. One common cause of death and other serious long-term complications after TBI is increased intracranial pressure (ICP) resulting from edema caused by the original injury. Increased ICP causes shift and deformation of brain tissue, complicating the injury further and rapidly proving fatal if unchecked. Cranial trepanation allows a monitoring device to be placed inside the skull, but puts the patient at risk of infection, bleeding, and further damage to the brain tissue. If possible, a non-invasive pre-screening method to evaluate the need for cranial trepanation is preferable. A CT scan is usually taken soon after TBI in emergency setting. It may show shifting of the tissue - "midline shift" - or a significant change in the size of the lateral ventricles. The shift and size of ventricles can either suggest for or against performing cranial trepanation. Surgeons may use both these features to evaluate the severity of the ICP, but these calculations have to be done manually and so involve estimation. In order to improve accuracy, speed and consistency, automated processing of this procedure is preferred. This paper proposes an method to automate the analysis of CT scan, including method of detecting ideal midline (where "ideal" refers to the position expected in a normal non-pathological subject), segmenting the lateral and third ventricles. The ventricle segmentation result can be used to provide statistics about the change of ventricles and for further diagnosis.

In this paper, bone symmetry and then direct detection of anatomical features in CT images are applied to capture the ideal midline. In capturing the midline, using symmetry alone cannot guarantee that the resulting line is consistent with the actual location of the ideal fissure (the fissure of the brain before TBI). In other words, while the fissure is a suitable representation of the midline, the fissure plane is not always representing the plane separating the two sides of the brain symmetrically. This is more evident in pathological cases associated with TBI where the midline is clearly shifted to one side, thus violating the symmetry of a typical normal brain. In [4], a genetic algorithm is applied to model the deformed midline; this may be the closest related work. This method assumes symmetric textures in the brain tissue. However, the algorithm does not work well when the midline shift is larger than 5 mm, which reflects the asymmetry present in most pathological cases.

**Figure 1:**
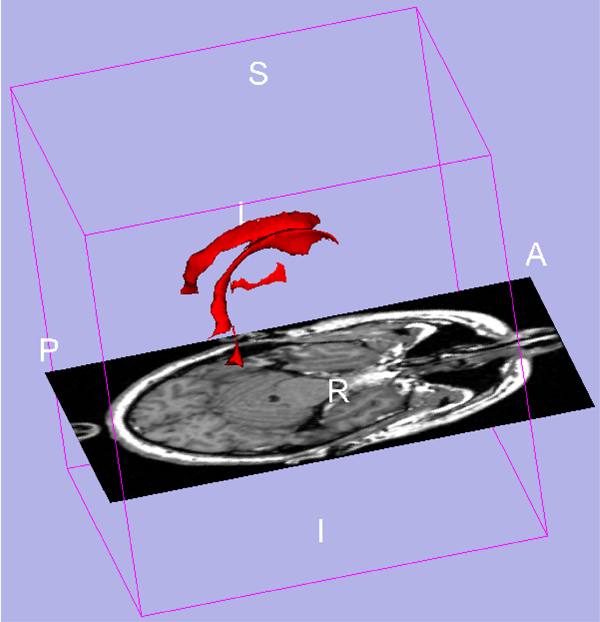
**3D ventricle model from 3D slicer sample visualization**. The red parts are ventricular system model. Two symmetrical lateral ventricles on each side in normal case. The lower part in the middle is the third ventricle. The fourth ventricle is the lowest part which is not considered in our paper, but it can be included following the method in this paper.

The ventricular system is the target for segmentation, as it is typically visible in CT scans and can be used for midline estimation. Figure 1 shows a 3D model of the ventricular system with the presence of a MR slice of the brain. In this paper, the fourth ventricle is not considered in this work because it is rarely used to measure the midline shift, but it can be easily included. Several automated segmentation techniques have been extensively specialized for medical images, particularly MRI scans. Segmentation methods for medical image can be divided into two groups based on their incorporation of prior knowledge. The first one uses prior/expert knowledge explicitly. One popular approach in whole brain MRI segmentation is the use of probabilistic anatomical atlas [5,6]. Region growing is another method of incorporating the continuity of objects in images, scans between adjacent slices especially in MRI. Xia et al. [7] apply this to extract ventricles from separated regions of interest in the brain, and design very specific rules to grow each part into the entire ventricle system. Region growing is also employed by Schonmeyer et al. [8] to segment lateral ventricles. It incorporates a descriptive feature rule based system, called cognition network technology, for image segmentation and shows promising results. The second group of segmentation methods applies classification and clustering methods, which can be considered as using prior knowledge implicitly through several training examples, or extracting some statistical features inside images to train a model. For example, artificial neural networks are used in [9] for segmentation of multi-spectral MRI scans. Wells et al. [10] use the expectation/maximization (EM) algorithm to classify white matter, gray matter. In [11], expectation maximization segmentation is combined with the use of active contour models. In [12], fuzzy c-means (FCM) clustering method is combined with bias field modeling for MRI segmentation.

However, in several of these studies the test subjects are healthy patients, or have relatively little distortion of brain structures. Furthermore, these studies all focus on MRI segmentation; research on automated brain CT segmentation is still relatively scarce. MRI offers improved soft tissue contrast over CT, and is therefore more capable of characterizing these tissues to facilitate segmentation [13]. However, while MRI is suitable for soft tissue examination and when time and cost are not important factors, in real clinical settings, e.g., in traumatic injuries, CT images are typically used due to time urgency, better visibility of bones and blood as well as low cost. This emphasizes the need to develop more effective methods for CT image processing. Though CT segmentation has been studied previously, much of the work has focused on cardiac and thoracic anatomy [14-16], for which CT is a standard method of diagnosis. In [17], a 2D atlas is used for segmentation of abdominal CT volume. Some attempts at segmenting brain CT images have been made. Li et al. [18] devised a method to model spatial relationships between objects in the brain. However, the algorithm was not tested on severe pathological cases. In [19] k-means clustering and neural network are applied to segment areas of hemorrhage in brain CT slices, but the approach is only tested on a single CT slice. The method described in this paper, besides using traditional image segmentation methods, also incorporates anatomical information, making the algorithm robust enough to deal with pathological cases. The two main challenges of segmenting axial head CT images are as follows: lack of high-resolution soft tissue definition compared to MRI and the difficulty of dealing with complex pathological subjects. In this paper, we present an algorithm on ideal midline detection and ventricular system segmentation to deal with these challenges. Testing is performed on a dataset of TBI cases provided by the Carolinas Healthcare System (CHS). The proposed segmentation method is validated on approximately 400 CT slices for multiple patients. The initial forms of the ideal midline detection and ventricle recognition algorithms were briefly described in our previous paper [20]. In this paper we present the details of our refined and extended algorithms.

## Methods

### Methodology overview

**Figure 2:**
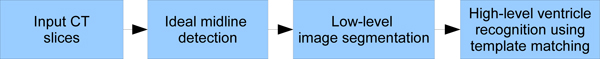
**The framework of ventricle segmentation**. This is the schematic description of our method. First ideal midline is detected and used to align CT scans in the same direction. Then low-level segmentation and high-level recognition are applied.

The high-level description of our processing of CT images is illustrated in Figure 2. The first step is to identify the ideal midline of the brain, as well as the angle of rotation of the skull versus the midline for calibration. This must be done prior to ventricle segmentation and recognition, since all slices must be correctly rotated to position the ideal midline in a vertical direction. This calibration step is vital for the success of template matching from MRI images later. Candidate ventricle objects are then identified in all slices via spatial segmentation techniques. These candidate objects are accepted or rejected using bounding boxes and template matching against a set of MRI scan slices. The shape and location of the lateral ventricles change over a sequence of slices, so each CT slice is mapped to the corresponding MRI slice to allow for appropriate variation in constraints on ventricle size and position. After the segmentation of ventricles, the actual midline is estimated based on feature points on the ventricles. The details of each step of the schematic diagram in Figure 1 are described in the following sections.

### Ideal midline detection

Increased ICP can cause the midline of the brain - the midpoint between the two lateral ventricles - to move from its original position. The detection of the ideal midline - i.e., the midline that should be present in a healthy brain with normal ICP - serves two purposes. The main reason is that it can be used as a reference line to measure the shift in the brain tissue. It can also be used as a calibration position, since different CT scans have different head rotation depending on patient position. Once calibrated based on the ideal midline, the whole anatomical structure of the brain can be roughly identified. This provides the opportunity to later use a template matching method for ventricle recognition. The ideal midline can be roughly approximated using the symmetry of the brain, but other features must be considered for more accurate detection. The method described in this paper uses the bone protrusion on the upper part of the skull and the falx cerebri fold in the lower part to accurately locate the position of the ideal midline, since these anatomical features change very little with midline shift in the brain. The ideal midline detection method has three steps:

1. Detect the approximate midline using symmetry.

2. Detect the falx-cerebri and anterior bone protrusion.

3. Use these features to refine the midline position.

When all slices in a CT scan have been processed, an adjustment is applied across the scan set to compensate for inaccurate midline detection in individual slices.

#### Detection of approximate position of the ideal midline using symmetry

**Figure 3:**
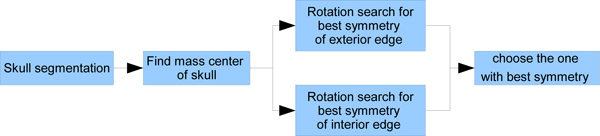
**Exhaustive symmetric position search**. This is the schematic description to detect approximate position of the ideal midline. First the skull is segmented because the intensity is much higher then other brain tissue. Then mass center is calculated and rotate the skull around it to find the best symmetric position. Both exterior edge and interior edge symmetry features are measured to find a better symmetry score.

**Figure 4:**
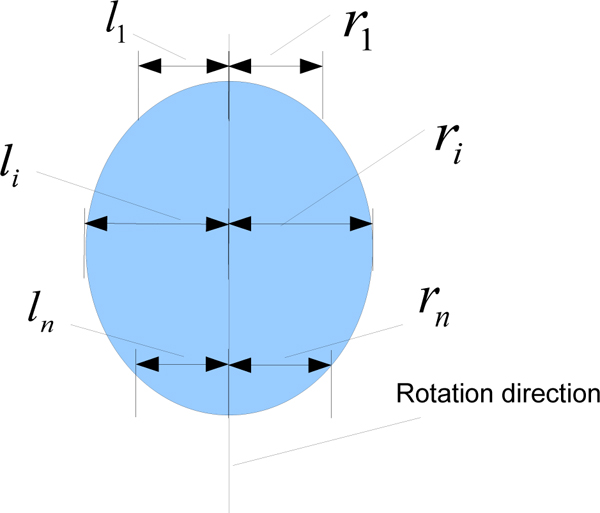
**Symmetry cost calculation**. The input is the edge of skull. The difference between distances from each side to the rotation center line is calculated and summed for all rows. The smaller the symmetry cost, the better the symmetry. The *l*_1 _and *r*_1 _actually begins at the top line of the skull (the ellipse), *l*_*n *_and *r*_*n *_are actually on the last row of the skull.

**Figure 5:**
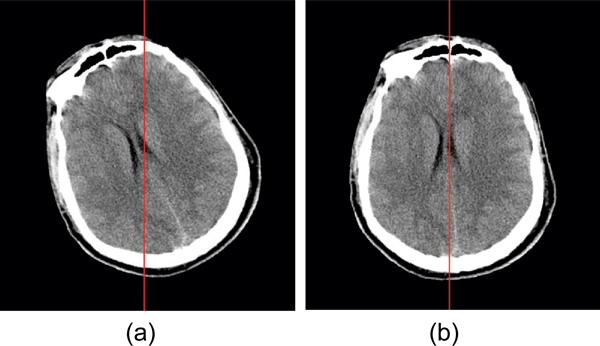
**Comparison of different symmetric measurements**. (a) This result uses the difference between thicknesses on each side of the skull to measure symmetry. (b) This result uses the difference between center-to-edge distances on each side. Because of the irregularity on the skull edge, the thickness based symmetry cost sometimes leads to wrong approximate position.

To find the approximate ideal midline, the algorithm uses an exhaustive rotation angle search around the mass center of the skull to find the line that maximizes the symmetry of the resulting halves. Figure 3 outlines this algorithm. First the bone image is extracted using a thresholding method. The image is then rotated around the center of mass of bone parts in the image. Finally, the symmetry of the resulting image is measured. Symmetry is defined here as the sum of the symmetry of each row in the resulting image. The row symmetry is defined as the difference in distance between each side of the skull edge and the current approximate midline. Figure 4 illustrates the symmetry measurement. For each rotation direction *θ*_*j*_, the symmetry cost *S*_*j *_is defined as follows:

(1)

where *n *is the number of rows for the segmented skull in the image and measures *l*_*i *_and *r*_*i *_are depicted in Figure 4. Our method resembles the method used in [4], except that we incorporate in our assessment both the interior edge of skull and the exterior edge of skull. Usually the interior edge is unaffected by the thickness of the skull wall, whereas using the exterior edge may prove problematic if the skull wall is not of uniform thickness. Figure 5 demonstrates such a case; the first image is the result of using thickness of skull bones for symmetry measurement, and the second uses the difference in distance from the searched line to each side. However, in some cases, especially in the lower part of brain, the presence of shape irregularities inside the skull makes the exterior edge a better choice for evaluating symmetry.

Consequently, both the interior and exterior edges are tested, and the edge with better symmetric measurement (the lower value of symmetric cost) is used.

Once an approximate midline is estimated using symmetry, brain anatomical features, i.e. the position of the falx cerebri and protrusion of skull bone are used to refine detection.

#### Detection of Falx cerebri at the lower part of the brain

**Figure 6:**
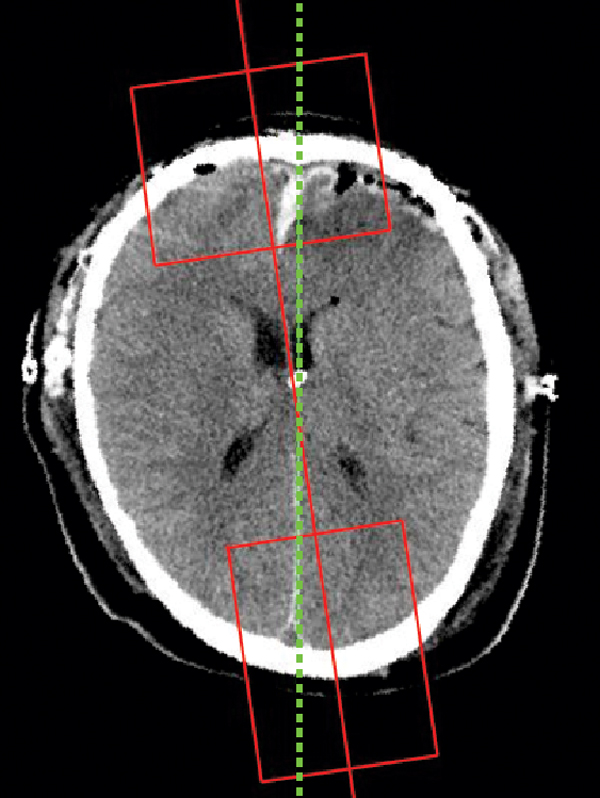
**Example midline detection result**. The green line is the final ideal midline detection result. The red line is the approximate line detected based on symmetry. Two red boxes are used to get the Region of Interest for protrusion and falx cerebri detection.

The falx cerebri is a strong fold of dura mater that follows the fissure between the left and right cerebral hemispheres. In order to detect this anatomical feature quickly and accurately, two searching rectangles are defined based on the ideal midline and the two intersection points with the skull bones. The size of the rectangle is chosen to include the anatomical features to be detected. All the feature processing steps are applied only in these cropped regions of interest (ROI). Figure 6 shows the two rectangle ROIs and refined ideal midline based on anatomical features. The method used to detect these features is explained in this and the following sections.

**Figure 7:**
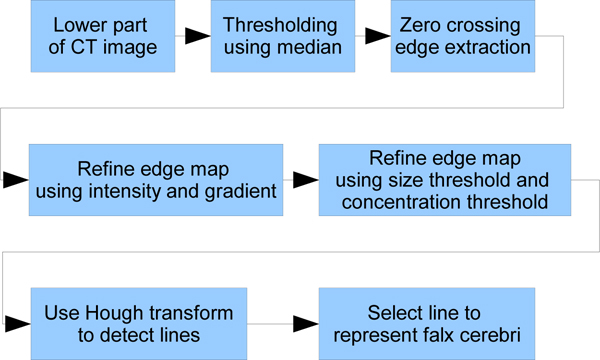
**Diagram of falx cerebri detection**. The input is the lower part of CT images selected by the rectangle box. Each step tries to filter irrelevant parts of the image and finally use Hough transform to select the line representing falx cerebri.

First, edge detection methods are used to extract the edge map. Hough transform is then applied to detect lines in the edge map. However, this only works if the gray line representing the falx cerebri is very obvious compared to the neighboring region. In some CT images, the area around the gray line has a similar gray scale pattern resulting in unwanted edges in the edge map. To address this issue and erase these edges, we first use a zero crossing edge detector to generate the edge map. This maintains richer edges during Sobel detection and allows removal of unwanted edges. The edge map is then refined step by step using known features of the falx cerebri. Finally, Hough transform is applied to detect lines representing the falx cerebri. Figure 7 shows the schematic diagram.

Zero crossing keeps nearly all the edges regardless of the gray scale values or the sharpness of an edge. Since the aim is to detect light gray lines, not the darker lines, we change all the gray scale values below the median value of the area to the median. After this step, only the much brighter pixels will retain their original gray levels in the image. This simplifies the edge map generated later. It was also discovered that the gray line representing the falx cerebri has sharper edges than most other boundaries, and this can be used as a guide to erase trivial edges. This information is incorporated by calculating the Sobel edge map and then applying a threshold to form a revised edge map. The threshold is set as a percentage of the maximal value in the Sobel edge map. Finally, only the edge points that appear in both maps are considered.

Another issue affecting the Hough transform is the presence of small unconnected point clouds close to edges. Hough transform may try to connect them and form a new line. This effect can be erased by removing all edge clouds with points smaller than some threshold value. In our experiment, we use 3 pixels as the threshold.

A final edge point erase is done by only selecting the vertical strips with high edge points along the entire vertical direction. The reason is that after obtaining the approximate position of the midline and rotating the CT images based on this, the gray lines in CT images are mostly vertical. The vertical strip containing the gray lines must therefore have more edge points than other places.

**Figure 8:**
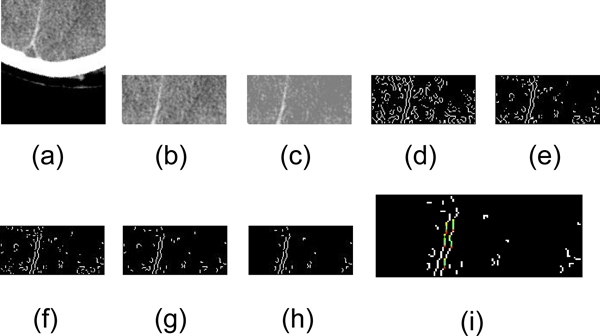
**Detection of lower falx cerebri**. (a) The lower part of CT image. (b) The lower part without skull. (c) Valley filling using median value. (d) Edge map after edge detection of zero crossing. (e) Edge map masked using intensity threshold on gray scale image. (f) Edge masked using gradient threshold on gray scale image. (g) Edge map after deleting small connected object. (h) Edge map after deleting sparse points part. (i) Hough Transform result on edge map.

After these filtering steps are applied to the edge map, we use the Hough transform to detect lines inside the map. Figure 8 displays the entire process. The results of the Hough transform are usually a set of lines. Two constraints must be established to extract the desirable lines from this set. First, the angle of the line must be in the range where the lines are concentrated. This range is obtained by calculating the statistics of the angles of the detected line. Second, the line must lie inside the range where the cluster of lines concentrates. The final ideal midline at this stage is chosen as the longest line satisfying the above constraints.

#### Protrusion detection

**Figure 9:**
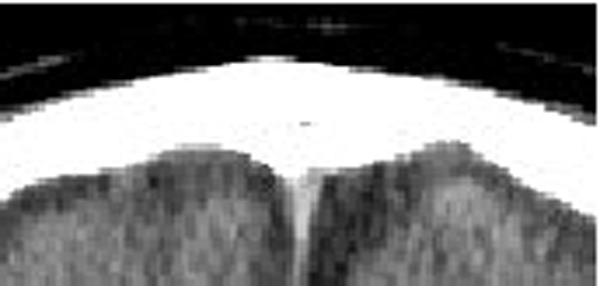
**The anterior bone protrusion**. This is the Region of Interest for bone protrusion detection. Most CT scans of the skull in this area show the protrusion feature.

A bone protrusion is located in the anterior section of the skull; the falx cerebri extends from this point. This anatomical feature can be used as a starting point for the midline. It can be seen in Figure 9 that the protrusion curves down to a local minimum point at the anterior edge of the falx cerebri.

**Figure 10:**
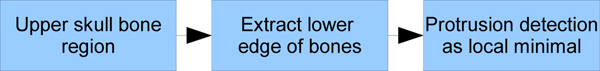
**Diagram of protrusion detection**. The interior edge of the skull is extracted based on the intensity. Then by viewing the extracted edge as a function curve versus the horizontal coordinate of the image, the protrusion is the local minimal point of the function curve.

If one views the lower edge of the skull bones as a curve on the image plane, then the curve can be modeled as a 1-dimensional function. Therefore, the protrusion point is the place where the function reaches its minimum. Detection of the protrusion point becomes a matter of finding the minimum of a sampled 1-dimensional function. There are two problems in real images which make this modeling more complex. One is that the protrusion location may not be the global minimum, i.e, the left and right edge may even be lower than the protrusion place. Therefore the height of the bone edge cannot be used directly to locate a local minima. Another problem is that there are small noisy local minima in other places. In order to address these problems, the derivative is used to find the local minima and a larger neighborhood is searched to avoid small noisy minima. Figure 10 explains protrusion detection graphically. Since the derivative is calculated in a discrete curve, obtaining a derivative of exactly zero may not be possible. A suitable alternative is to find the transition point between a negative and positive derivative. Due to the uneven surface of the skull, there may be more than one local minimum inside the region of interest, so the algorithm uses the signed addition of derivatives within a specified neighborhood of the local minimal point. This gives significant preference to large minima over the smaller local minima that occur naturally on the skull surface. If there are multiple large minima in the region of interest, the one closest to the approximate symmetric midline is chosen.

**Figure 11:**
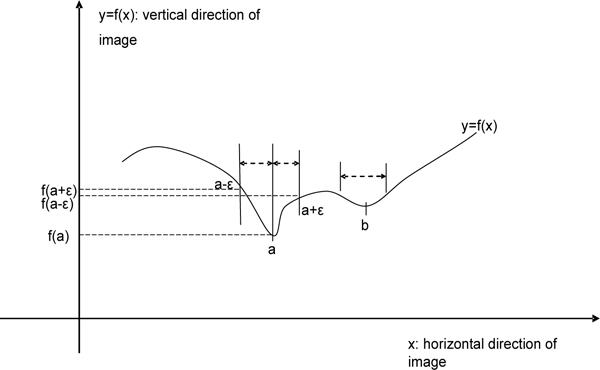
**Selecting a larger protrusion**. Function *f*(*x*) is the extracted curve of the interior bone edge. Because of the noise and irregularities in the skull, there are several small local minimal points on the extracted edge. In order to select the largest one, second derivative is used for comparison.

Figure 11 shows the curve of a typical bone shape. The aim is to find the point *a *in the presence of multiple local minima. The difference between point *a*, the true minimum, and *b*, the small protrusion due to irregularity of bones, is the size of the slope around them. This can be captured by increasing the size of the neighborhood studied when calculating the derivative; typically the algorithm uses a region of 10 - 15 pixels. The point *a *is found by searching the maximum of the sum of the left and right derivatives as follows:

(2)

(3)

where *w *is the neighborhood width. Equation 3 finds the maximal second derivative. This calculation avoids small amounts of noise around the curve.

#### Post-processing

**Figure 12:**
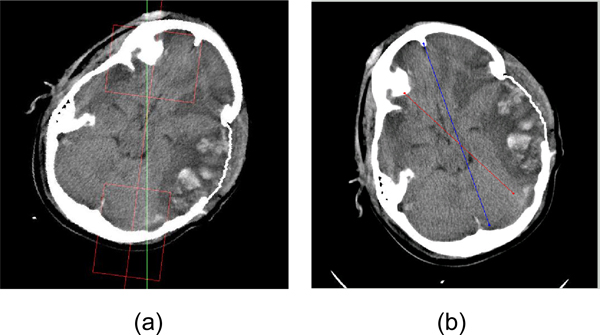
**Consistency check**. (a) The slice with incorrect midline detection. (b) the corrected slice, where the blue line shows the new midline position.

According to the opinion of participating physician, the midline detection at this stage is accurate in over 90% of the slices where both the anterior bone protrusion and the falx cerebri line are present. In slices where one of these features is not present, the symmetric exhaustive rotation search is repeated with a smaller range and smaller degree steps around whichever feature is present. If neither feature is present, the initial approximate midline may be used; however, since the skull shape is sometimes complex, the symmetry of the skull will not reflect the true midline. In such cases, in our algorithm, slice comparison solves the problem. From our experiment, it is observed that inaccurate midline detection seldom occurs in consecutive image slices, and it is reasonable to assume that the midline should not change much across several consecutive slices of the scan. Using this knowledge, a slice with an "abnormal" midline can be corrected based on the midlines of the slices immediately preceding and following. The adjustment initially identifies the "first class" slices - those with both features detected - then the "second class" slices - those with only one or none feature detected. Then it calculates the angle difference between each second class slice and its closest first class neighbor. If the difference exceeds a certain threshold, the midline is deemed unacceptable, and is replaced with the midline of the closest first class neighbor. The threshold is chosen after some statistical analysis of data observed. This post-processing has two benefits: to guide later segmentation, and to lay a basis for the calculation of structural information after segmentation is complete. Figure 12 shows the result of the adjustment based on neighbor slices.

### Low-level segmentation

In CT scans, the ventricle system typically has darker color than other tissue. A simple method to segment the ventricle would be using binary thresholding, with the threshold value chosen via Otsu's method [21]. However, testing this method gave poor results, with severe noise and object fragmentation. The challenge is that in CT scans, there are a lot of "holes" or noise (which has different gray scale values) in tissue areas. Another challenge is that the boundary between different tissue parts is very blurry. Binary segmentation is also unrealistic since there are multiple tissue types within the brain. Furthermore, simple thresholding classifies each pixel independently of its neighbors. This cannot work well when different tissues have wide range of overlap of gray scales. To address the problem of noise and blurry boundary, segmentation methods adopted here follow conditional probability models where the most probable label of a pixel depends upon the attributes of both the pixel itself and its neighbors. In this paper, two such spatial segmentation methods are evaluated: Iterated Conditional Models (ICM) and Maximum A Posteriori Spatial Probability (MASP) [22].

**Figure 13:**
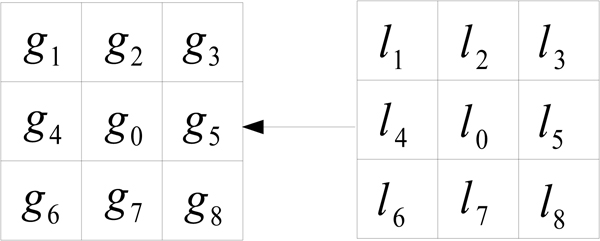
**Gray scale grid and label grid of images**. The model assumes that the gray scales in each image is generated by its labels using some mechanism, e.g., using sampling from Gaussian distribution. There may be some dependency among labels, e.g., local dependency modeled by Markov Field.

For both methods, some starting assumptions are made. It is assumed that each pixel *x *in an image **X **has an attribute *l*_*x *_representing the label to which it is assigned, and an attribute *g*_*x *_representing its gray-level intensity. This gives rise to two subimages **L **and **G**, where **L **= *l*_*x*_, *x *∈ *X *and **G **= *g*_*x*_, *x *∈ *X*. **G **is the observable image of gray-level values, and **L **is the 'hidden' image of assigned labels. The labels in **L **each take some value *k *∈ *K*, where *K *is the number of distinct objects (or tissue types) in the image. Except for those on the image border, each pixel *x *has M neighboring pixels, and these form a neighborhood *N*_*x *_= (*x*^(1)^, *x*^(2)^,..., *x*^(*m*)^). This is split into a neighborhood label configuration , and a neighborhood gray-level configuration . The set of all possible neighborhood configurations is denoted by **N**. Usually a 4-neighborhood or 8-neighborhood are used. Figure 13 shows the 8-neighborhood gray-scale block and label block in an image. Generally, in our application, four types of brain matter - bone or blood, ventricular tissue, and light and dark grey matter - are assumed to represent different ranges of gray scale in the brain.

In using the spatial information in each pixel's neighborhood, two assumptions are made. First, that the gray scale value *g*_*x *_of pixel *x *depends only on the label of *x *and not its gray-scale neighborhood, i.e., *g*_*x *_and *N*_*g *_are independent given the label *l*_*x*_. Second, the dependence between the labels are local. ICM and MASP have different assumptions on local dependence.

#### Iterated Conditional Modes (ICM)

ICM [23] follows the two assumptions made about spatial segmentation: a pixel's gray scale value *g*_*x *_is dependent only on its label *l*_*x *_and not the values of other pixels given the label *l*(*x*); the ideal label map L* is a realization of a locally dependent Markov Random Field with distribution *p*(*x*). The statistics of each label class are computed using the Gaussian model, so each class has parameter set the mean *μ*_*k *_and the variance *σ*_*k *_which can be estimated from the image. Therefore the conditional density of the image *G*_*x *_given *L*_*x *_is given by the following due to independence assumption.

(4)

ICM iteratively finds a local maximum of *P*(*L*_*x*_|*G*_*x*_), updating each pixel's label in every cycle, which can be expressed in the following Equation:

(5)

Suppose that  is a provisional estimate of the true label map L*, and at each step the estimated  is updated at each pixel *x *given all available information. For each pixel *x*, denote *x *= *k *meaning that pixel *x *has label *k, k *= 1 ⋯ *K*. Denote *g *the gray-scale value of *x*, *N *as one of the neighborhood labels configuration of *x *and *N*_*c *_the current estimation of neighborhood configuration, which is from . *G *is the gray-scale values of *x'*s neighborhood. By using the two assumptions, Equation 5 can be written as

(6)

where *f*(*g*|*x *= *k*) is the probability of a pixel *x *having a particular gray-scale value *g *given its label *k*, and *p*(*x *= *k*|*N*_*c*_) is the probability of being label *k *given the labels of its current estimated surrounding neighbors *N*_*c*_. A single cycle applies this to each pixel in turn, and the cycle is repeated until the estimated label map  does not change significantly between iterations.

Note that the ICM method requires initial segmentation of the CT image. Here k-means is used, due to its speed and reasonably accurate performance. The initial seeds are chosen manually, after using training examples to calculate a standard gray scale range for each type of brain tissue. These ranges are consistent across the majority of images, and k-means initialization is therefore satisfactory.

#### Maximum A Posteriori Spatial Probability (MASP) segmentation

As in ICM, there are also two assumptions in the MASP [22] segmentation algorithm. First is the same independence assumption about the gray-scale value given the label of the pixel. Second is about local dependence of labels. The neighborhood configuration depends on which class or label the center pixel belongs to. Using the same notation as in ICM section, MASP tries to label each pixel using the following formula:

(7)

where

(8)

Since *P*(*g*, *G*) is independent of the label *k*, we have

(9)

Calculating *P*(*g*, *G*|*x *= *k*) requires more effort. By using the law of total probability, *P*(*g*, *G*|*x *= *k*) can be used to calculate as follows:

(10)

which, using assumption 1, can be written as:

(11)

It also assumes that *f*(*g*|*x *= *k*) follows a normal distribution with parameters *μ*_*k*_, the mean grey-level for class *k*, and *σ*_*k*_, the standard deviation of the gray-scale values for class or label *k*.

#### Modified MASP

In this section our modified version of MASP, used in this paper, is described. The computational demands of the conventional MASP process can be significant. Since the algorithm averages probabilities over all possible neighborhoods in Equation 11, its dimension increases rapidly with more complex images. A simple binary segmentation using a 4 pixel neighborhood has a dimension of only 2^4 ^= 16. However, CT image segmentation is more complicated for two reasons. Firstly, at least 4 labels are required: bone, ventricle tissue, and light and dark grey matter. Secondly, as mentioned before, if we use an 8-pixel neighborhood, the resulting dimension is therefore 4^8 ^= 65536 which is a very large number for probability estimation. Moreover, the MASP algorithm is applied iteratively to each individual CT slice, taking around 8 iterations on average, and each slice contains 512 × 512 = 262144 pixels, with up to 8 slices per patient scan. All these result in very long computation time for MASP. Since speed is vital when medical decisions must be made quickly, this high dimension results in unacceptably slow performance. In [24], we proposed that instead of averaging the probabilities over all neighborhood configurations, the current estimation of labels can be used. The modified MASP algorithm uses only the current estimated neighborhood for the each pixel. In this case, instead of calculating *P*(*g*, *G*|*x *= *k*), it actually calculates *P*(*g*, *G*, *N*_*c*_|*x *= *k*) as follows:

(12)

From the independence assumption, we have

(13)

(14)

The modified algorithm significantly improves the segmentation speed for each individual slice, with no obvious negative impact on performance.

### High-level template matching

**Figure 14:**
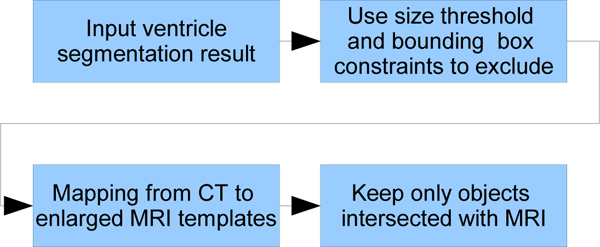
**Diagram of ventricle recognition**. This is the schematic diagram of ventricle recognition. First size and location constraints are used to exclude segmented areas of non-ventricle parts. Then by mapping the template to the segmented image, more precise exclusions base on location can be achieved.

After initial low-level segmentation, the image pixels are grouped into different types of tissue. Since initial segmentation only relies on gray scale and highly local neighborhood information, this leads to poor performance on pathological CT images, as they may contain bruised tissues which are very similar to ventricle matter in terms of local neighborhood intensity statistics. However, the use of templates can improve the discrimination between the two different tissue types by considering the location as a feature. The schematic diagram of ventricle identification from initial segmentation result is shown in Figure 14. First, a size threshold is applied to all candidate ventricle objects to remove noise and small artifacts. In experimental results, it was observed that non-ventricular objects that resemble ventricles, i.e. a bruise area, often appear very close to the edge of the brain. Two bounding boxes are therefore used to exclude these objects; one large and one small, both centered in the middle area of the skull. The small box applies the constraint that all ventricle parts should be inside a certain area of the brain; the large box applies the constraint that all ventricle parts should not exceed a certain area bound. If some portion of a candidate object falls outside of the large bounding box, or some portion does not fall within the small bounding box, the object is rejected. The sizes of the two boxes were determined via experimental analysis on a training set of CT images.

**Figure 15:**
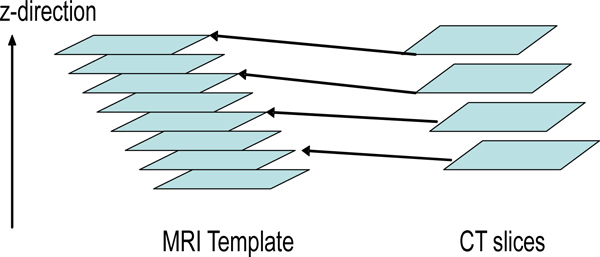
**Mapping between MRI template and CT slices**. The z-direction is the direction from lower part slice to upper part. Since MRI template slices are denser than CT scans in the dataset, CT slices are mapped to corresponding MRI slices using linear mapping. In most cases, this mapping is satisfactory.

**Figure 16:**
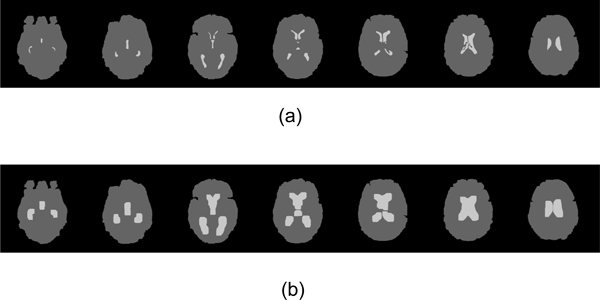
**MRI template set after dilation**. (a) The bright parts are the original MRI template including two lateral ventricles and third ventricles. The gray parts are the brain parts. (b) In order to accommodate the variations of ventricle positions in pathological cases, the template slices are enlarged by morphological dilation operation.

Though the bounding boxes successfully reject most non-ventricular objects, some are too close to the true ventricles to be excluded. Since the shape and position of the ventricles changes across the multiple slices in a full CT scan, if the position of the current slice in the sequence is known then more precise constraints can be used. For example, provided the full CT scan set is available, the location of a specific CT slice in the brain can be determined based on the resolution of the direction perpendicular to the axial plane (referred to as the *z *direction) and the order of the slice. The brain atlas information can be used to obtain the ventricle shape information at that specific location. The algorithm in this paper use a brain MRI template set. The template is generated based on the annotated images from the Digital Anatomist Interactive Atlas of University of Washington, . to extract ventricle shape information. The fourth ventricle is not considered in this work because it is rarely used to measure the midline shift, however the same principle can be applied for the fourth ventricle recognition. Due to the resolution difference between CT scan and MRI scan, a slice order mapping between these two sets are required. Figure 15 illustrates this mapping. Once the mapping of a CT slice to an MRI ventricle template is known, it can be applied for the majority of all other CT slices. While the resolution of the CT scan depends on the CT machine type and settings, the resolution for a given set of scans is fixed. In addition, the ventricle systems in the *z*-direction do not show significant variation across the training set. Therefore, mappings can be approximated with linear form. In the method described here, these mappings are first initialized manually and then optimized using a set of training images. Due to the slight variance in ventricle position as well as ventricle deformation across multiple patients, the original MRI template is enlarged via morphological dilation. The enlarged template provides an estimation of the position of ventricles. Figure 16 shows the enlarged MRI templates.

**Figure 17:**
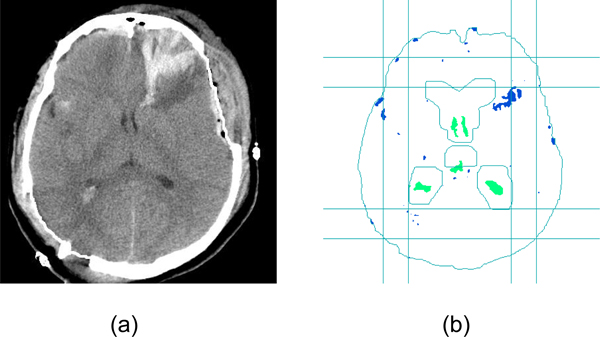
**Results of ventricle detection**. (a) This is the original CT image. (b) The straight lines are two bounding boxes constraints. Size threshold are also used to exclude small parts. It also shows the outline of the template shape. The segmented objects intersected with the enlarged template are recognized as ventricles.

After selecting the specific MRI template through slice mapping between CT scan and MRI scan, the template is aligned with the CT slice. The alignment is largely simplified by using calibrated CT scans from the ideal midline detection step, because there is no need to rotate images for alignment. The template is resized to make sure that the bounding box of the MRI template (which is the minimal rectangle covering the object) and the bounding box of the CT scan are the same size. Any candidate segment that intersects with the template is accepted as ventricular object. Figure 17 shows an example of the ventricle recognition step. From the results, one can see that the ventricle parts are successfully recognized using size, bounding box and template constraints.

The advantage of the proposed ventricle detection method over existing image segmentation approaches is that it incorporates anatomical knowledge into the detection procedure such as in the detection of the ideal midline. In ventricle recognition, prior information is embodied in spatial templates derived from MRI images.

## Results

### Data

The testing CT dataset was provided by the Carolinas Healthcare System (CHS). All subjects were diagnosed with mild to severe TBI when first admitted to hospital. The dataset contains 40 patients. The age range is from 8 to 70, with average age 38.4 and standard deviation of 19. From this set, 391 axial CT scan slices are selected that show ventricles or region that should have contained ventricles. To our knowledge, and based on the literature of the brain CT image segmentation [18,19,25,26], our database is relatively large, and as a result, the reliability of the testing results for the proposed CT segmentation method should be at least as high as that of almost all comparable works in this field.

### Results of ideal midline detection

The midline detection algorithm was tested on 391 CT slices. Out of these, 291 were detected as first type slices, and 100 were detected as second type. Performance was evaluated by the collaborating physician, as this is the only method to identify the true ideal midline and compare it to the one detected by the algorithm. Prior to the inter-slice consistency check, the accuracy for first class images was approximately 90%. With the consecutive inter-slice adjustment added, the accuracy increased to 98%, more over 95% of the detected midlines in the second class images were also acceptable. An example result is shown in Figure 6. The solid line is the initial midline approximation and the dashed line represents the refined ideal midline.

### Analysis of ICM and MASP

Since both ICM and MASP use spatial neighborhood information and probability model in their algorithm, it is interesting to compare these two algorithms. In the following we analyze the similarity, differences and their link between these algorithms.

#### Similarity

One of the two assumptions in ICM and MASP is the same, which assumes that given the label of the pixel, its gray scale value is independent of other neighborhood labels. For the other assumptions in ICM and MASP, the common points are that both assumptions assume local neighborhood dependence. In sum, both methods assumes a two layer way of viewing images. The higher layer is the label grid for each pixel location, the lower layer is the actual image based on Gaussian distribution for each label.

#### Differences

The ICM assumes Markov Random Field model to generate the label grid. This means that the model is expressed as *p*(*x *= *k*|*N*), where *x *is the center label and *N *is the neighborhood labels configuration. MASP, however, assumes that the neighborhood depends on the center pixel, which can be expressed as *p*(*N*|*x *= *k*). The directions of dependence is the main difference between these two methods. Other differences are the calculations made to estimate these probabilities. In ICM, *p*(*x *= *k*|*N*) is calculated using a defined probability function, e.g., using ratio of labels in the neighborhood to calculate the probability function. In MASP, the probability *p*(*N*|*x *= *k*) is estimated from the image by counting the proportion of each pattern. If the number of distinct labels *K *is large, MASP is computationally expensive because the number of neighborhood pattern is *K*^*n*^, where *n *is the number of pixels defined in neighborhood.

#### Relationship between ICM and MASP

It can be proved that the objective functions used in modified MASP and ICM are equivalent, the only difference is the different approaches to evaluate conditional probability functions based on the different assumptions made in the two cases. For ICM, the probability function being evaluated is

(15)

Modified MASP attempts to maximize the following function with different label *k*:

(16)

(17)

(18)

(19)

(20)

As it can be seen, in terms of formal expression, both ICM and modified MASP try to maximize the probability of *P*(*x *= *k*) conditioned on its gray scale *g *and its current estimated neighborhood *N*_*c*_. The difference between modified MASP and ICM are that ICM use MRF to model the probability function *P*(*x *= *k*|*N*_*c*_), which is later estimated using current estimated labels, where as modified MASP uses the current estimated labels directly to estimate the probability *P*(*N*_*c*_|*x *= *k*). Since they both use the same objective function to choose labels, ICM and modified MASP should have similar labeling result. However, because of the large space of neighborhood configuration, using image data for probability estimation may not be sufficient. Therefore ICM seems a safer method of using spatial information.

#### Illustration on CT Ddta

**Figure 18:**
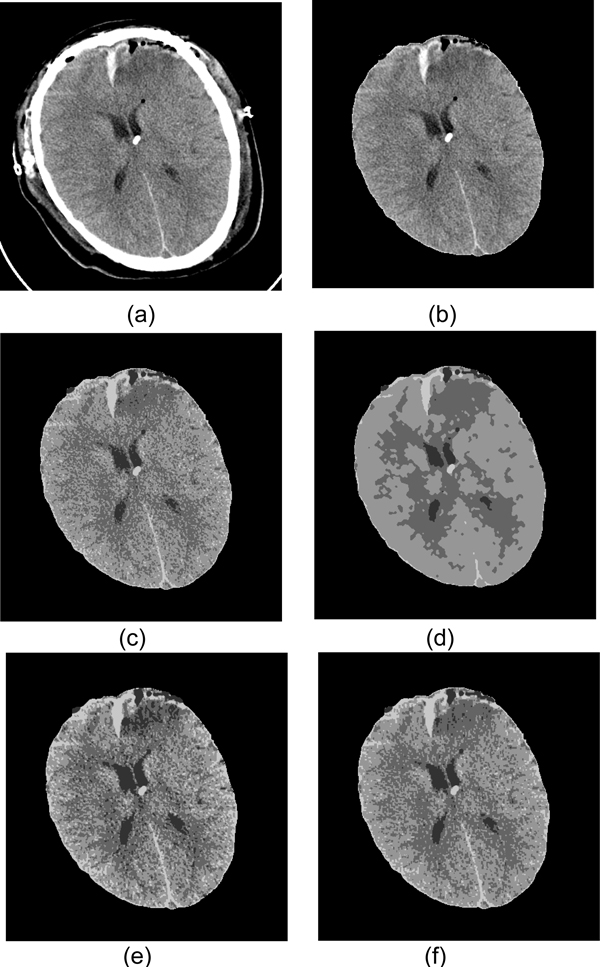
**Comparison of Segmentation methods**. (a) Original CT Image. (b) CT Image without skull. (c) K-means clustering result. Intensity value for each pixel is used as the feature. Four clusters are specified with initial seeds. The problem for K-means is that there are holes in the result segmentation because of uneven intensity distribution for the same part as well as noise. (d) ICM segmentation result with K-means segmentation as initial result. It result smoother result, although it lose some small parts, like the tip of left horn. (e) MASP segmentation result. This result has more noise than ICM. For example, the some parts of bruise area on the right upper corner are labeled as ventricle part. (f) Modified MASP segmentation result. This result is in the middle between ICM and MASP. It has less noise than MASP but not as smooth as ICM, which supports our mathematical analysis.

In order to assess and compare the performance of the segmentation algorithms, we apply both methods on the CT dataset. For ICM, the simplest non-degenerated MRF is used and *β *= 1.5 [23]. For MASP and modified MASP, 8-neighborhood is used. Each algorithm runs for 10 iterations, given a starting label map generated via k-means with fixed seeds initialization. As a baseline, K-means result is also presented. The difference between K-means, ICM, MASP and modified MASP can be seen in Figure 18. From the result we can see that ICM, MASP and modified MASP have smoother effect than simple K-means. This is due to the incorporation of local neighborhood information into labeling pixels. Compared to MASP, ICM has very clear boundary and shows a suitable performance dealing with noise. However, due to the smoothing effect, ICM also loses some detailed information, e.g., the upper horn on the lateral ventricles. MASP tends to maintain more details in segmented result, however it also keeps noisy part inside. For example, on the upper right corner, a part of the bruise region is classified as ventricle class. Since modified MASP has similarity with both ICM and MASP, it shows intermediate segmentation result, i.e., it shows more noise than ICM while keeping some detail of horns. However, it has less noise than MASP. Testing results on other CT images show similar characteristics of these algorithms.

### Results of ventricle segmentation

Since ICM is faster and has smoother results than MASP, ICM is used as our low-level segmentation method for evaluation of the proposed entire ventricle segmentation method. Then template matching method is used to recognize ventricles. Evaluation of final ventricle recognition was done by visual inspection conducted by the participating physician. Here two measurements are defined to evaluate the result. The first is a sensitivity-like measure. It is the rate of slices in which ventricular regions are correctly detected as ventricle. The second is a false positive-like measure. It is the rate of slices in which non-ventricular regions are incorrectly detected as ventricle. For the first measurement, the rate is 100% because all ventricles are identified in all slices. The false positives-like measurement is 8.59%, i.e., 33 out of 391 slices have falsely classified ventricles. These false positive objects in CT slices either included non-ventricle objects, e.g. bruised areas, or detected objects in slices where no ventricles were originally visible, which implies some mismatch between the adopted segmentation method and segmentation based on physician's interpretation.

## Conclusion and future work

This paper provides a method for automatic ventricle segmentation as well as an ideal midline detection algorithm using axial head CT images. First, an accurate ideal midline detection method is proposed, and used to correctly align the scan set based on the ideal midline. This is a crucial step before using any template matching later because it avoids the need to shift or rotate templates, and therefore simplifies registration. Then low-level segmentation and high-level ventricle recognition using template matching are applied. Our method offers the following improvements over existing approaches: accurate detection of the ideal midline, ventricle recognition using both anatomical features and spatial templates derived from MRI images. In addition, ICM and MASP are analyzed and compared both mathematically and experimentally. The relatively large size of the CT dataset used for testing makes the results of this study more reliable. Future work will focus on refinement of these algorithms and the use of the extracted features to calculate changes in ventricle volume as well as midline shift based on the segmented ventricles, which may lead to a mapping between midline shift and increased ICP in patients with traumatic brain injuries.

## List of abbreviations used

(TBI): Traumatic Brain Injury; (MRI): Magnetic Resonance Imaging; (CT): Computed Tomography; (ICP): intracranial pressure; (ROI): regions of interest; (ICM): Iterated Conditional Models; (MASP): Maximum A Posteriori Spatial Probability

## Competing interests

The authors declare that they have no competing interests.

## Authors' contributions

WC participated in the algorithm design on ideal midline detection, high level template matching, ICM part, analysis of ICM and MASP and drafted the manuscript. RS participated in the MASP part, segmentation result comparison, discussion of algorithms, drafted and revised the manuscript. SJ participated in design of detection of falx cerebri and coordination. KW participated in the evaluation of experiment results and guide of CT image features to be extracted. KN participated in design of the algorithms, revision of manuscript.
